# Strategic differentiation and integration of genomic-level heritabilities facilitate individual differences in preparedness and plasticity of human life history

**DOI:** 10.3389/fpsyg.2015.00422

**Published:** 2015-04-22

**Authors:** Michael A. Woodley of Menie, Aurelio José Figueredo, Tomás Cabeza de Baca, Heitor B. F. Fernandes, Guy Madison, Pedro S. A. Wolf, Candace J. Black

**Affiliations:** ^1^Department of Psychology, Technische Universität ChemnitzChemnitz, Germany; ^2^Center Leo Apostel for Interdisciplinary Studies, Vrije Universiteit BrusselBrussels, Belgium; ^3^Department of Psychology, University of ArizonaTucson, AZ, USA; ^4^Department of Psychiatry, School of Medicine, University of California, San FranciscoSan Francisco, CA, USA; ^5^Department of Psychology, Federal University of Rio Grande do SulPorto Alegre, Brazil; ^6^Department of Psychology, Umeå UniversityUmeå, Sweden; ^7^Centre for Social Science Research and Department of Psychology, University of CapetownCapetown, South Africa; ^8^Department of Psychology, University of ArizonaTucson, AZ, USA

**Keywords:** heritabilities, transmissabilities, SD-IE, plasticity, preparedness

## Abstract

Life history (LH) strategies refer to the pattern of allocations of bioenergetic and material resources into different domains of fitness. While LH is known to have moderate to high population-level heritability in humans, both at the level of the high-order factor (Super-K) and the lower-order factors (K, Covitality, and the General Factor of Personality), several important questions remain unexplored. Here, we apply the Continuous Parameter Estimation Model to measure individual genomic-level heritabilities (termed transmissibilities). These transmissibility values were computed for the latent hierarchical structure and developmental dynamics of LH strategy, and demonstrate; (1) moderate to high heritability of factor loadings of Super-K on its lower-order factors, evidencing biological preparedness, genetic accommodation, and the gene-culture coevolution of biased epigenetic rules of development; (2) moderate to high heritability of the magnitudes of the effect of the higher-order factors upon their loadings on their constituent factors, evidencing genetic constraints upon phenotypic plasticity; and (3) that heritability of the LH factors, their factor loadings, and the magnitudes of the correlations among factors, are weaker among individuals with slower LH speeds. The results were obtained from an American sample of 316 monozygotic (MZ) and 274 dizygotic (DZ) twin dyads and a Swedish sample of 863 MZ and 475 DZ twin dyads, and indicate that inter-individual variation in transmissibility is a function of individual socioecological selection pressures. Our novel technique, opens new avenues for analyzing complex interactions among heritable traits inaccessible to standard structural equation methods.

## Introduction

The purpose of this paper is threefold: (1) to introduce the use of the Continuous Parameter Estimation Method (CPEM) to enable the estimation of genomic-level heritabilities, which we call individual *transmissibilities*, in population-representative samples from Sweden and the USA; (2) to apply this method to estimate the aggregate heritabilities of the upper two strata of the hierarchically-organized latent structure of Life History (LH) strategy; and (3) to extend this method further to estimate the heritabilities of the dynamics of Strategic Differentiation-Integration Effort (SD-IE) that have been previously documented among the different levels of the latent LH hierarchy (Figueredo et al., 2013b). Although the second objective has already been achieved by more conventional means (see Figueredo et al., 2004; Figueredo and Rushton, 2009), the third objective is not attainable without the novel method used to perform the first objective. Executing the second step, however, determines whether this novel method performs as expected in approximating the results previously achieved by more traditional means.

### Life history theory: an overview

Life history theory describes the pattern of allocations of material and bioenergetic resources into different domains of fitness in response to levels of environmental stability, extrinsic morbidity-mortality, and population density (Ellis et al., 2009). Mammalian life history strategies in general, including human ones, cohere into a continuum that varies from fast to slow (Promislow and Harvey, 1990; Ellis et al., 2009; van Schaik and Isler, 2012). Faster life history strategies are characterized by rapid ontogenetic development, early reproduction, high mating effort, and slower life history strategies are by comparison characterized by more parental effort, community building, and greater longevity (MacArthur and Wilson, 1967; Pianka, 1970; Rushton, 1985).

**Figure 1:**
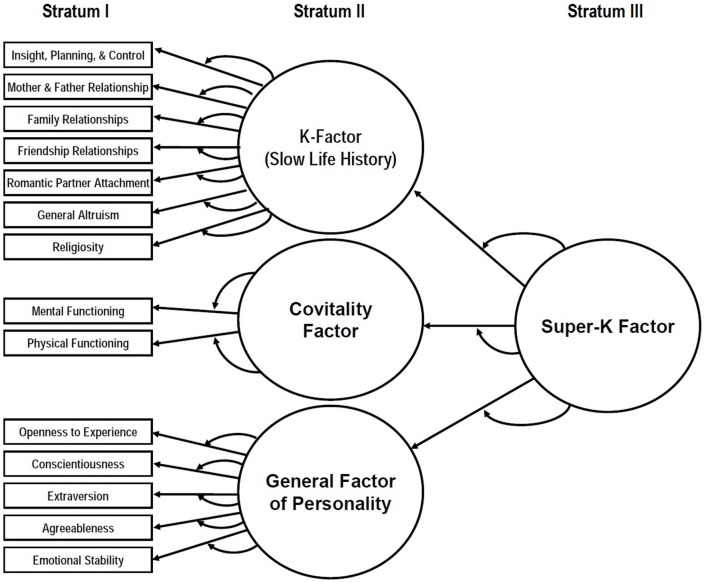
**The hierarchically-organized latent structure of life history traits**.

The latent structure of life history strategy was first extended by Rushton (1985) to encompass additional psychosocial traits in human populations, including a theoretically-specified profile of personality traits (see Figueredo et al., 2013a). This latent structure was later found to be hierarchically organized, with a higher-order factor, termed *Super-K*, at the apex of this hierarchy (Figueredo et al., 2004, 2007; Figueredo and Rushton, 2009). By analogy with the three stratum theory of human intelligence (Carroll, 1993, 1997), this apex can be designated Stratum III. Stratum II is populated by at least three lower-order factors so far identified: (1) the *K*-Factor, which relates to various measures of altruistic dispositions toward family of origin, long-term pair-bonding, parental investment, nepotism toward extended kin, altruism toward the community, and an orientation toward conventional religious piety (Figueredo et al., 2004, 2007); (2) the *Covitality Factor*, which relates to manifestations of physical and mental health which are in turn an outcome of higher levels of somatic effort (Weiss et al., 2002; Figueredo et al., 2004, 2007); and (3) the *General Factor of Personality*, or *GFP* for short, underlying the conventional personality dimensions, such as the “Big Five” personality traits, collectively forming a global measure of prosocial orientation and social efficacy (Figueredo et al., 2004, 2007; Musek, 2007; Rushton and Irwing, 2011). Stratum I is populated by a variety of more narrowly focused psychometric scales measuring domain-specific resource allocations within these broader common factors. This three-stratum hierarchy of latent life history traits is depicted graphically in Figure 1.

### Strategic differentiation-integration effort

The two fundamental requirements for a robust science of life history evolution are (i) methods of measuring heritable variation in life history traits within populations, and (ii) a theory describing how the variation in life history traits evolves (Lande, 1982). Advances on these fronts are facilitated by better understanding of the phenotypic and genetic associations between life history related sources of phenotypic variance. Consistent with this, it has been demonstrated that the phenotypic correlations among life history traits are stronger among individuals (and groups) exhibiting faster life histories than among those with slower ones. This phenomenon has been termed strategic differentiation-integration effort (SD-IE), and has been consistently demonstrated in various samples at the level of both individual (Figueredo et al., 2013b) and group differences (Fernandes and Woodley, 2013; Armstrong et al., 2014; Dunkel et al., 2014; Woodley and Fernandes, 2014; Woodley et al., 2014). Slower life histories appear to be more differentiated among themselves, possibly due to environmental predictability and heightened social competition at the carrying capacity during their evolution, which theoretically encourages specialization into various stable socio ecological micro-niches through intraspecific character displacement (Woodley, 2011; Figueredo et al., 2013b). Conversely, individuals and groups exhibiting comparatively faster life histories appear to be more similar among themselves, as they exhibit generalized tactics and need to be able to contingently switch between socio ecological micro-niches that are insufficiently stable for specialization—a common feature of an evolutionary ecology where the environmental stability is expected to be low (Woodley, 2011; Figueredo et al., 2013b). In Figure 1, the curved, single-headed arrows going from the latent common factors to the straight, single-headed arrows connecting those with their manifest indicators (i.e., the factor loadings) represent the moderating SD-IE effects, and have been found to be generally negative in direction (i.e., the strength of the associations weaken as the level of the latent trait increases), with only a few theoretically-expected exceptions.

Also important to SD-IE are the concepts of *preparedness* and *plasticity* (Figueredo et al., 2006). The former represents the degree to which an organism is genetically predisposed toward a particular developmental trajectory, whereas the latter constitutes the degree to which gene-environment interaction induced phenotypic changes during development may alter that prepared trajectory. Both of these principles relate directly to the concepts of genetic accommodation (West-Eberhard, 2003), which encompasses that of genetic assimilation (Waddington, 1953). It has been argued that, just as selection can favor the predisposition toward a particular developmental trajectory in ontogeny under conditions where this confers increased fitness, certain selection regimes might instead confer fitness on the ability to generate a highly plastic but persistent phenotype, where different phenotypes can be developed by the same genotype under different but stable ecologies. Depending on the rate of environmental variability over time, a shorter-term form of plasticity, called *flexibility*, describes the capacity for an organism to alternate rapidly between different micro-niches, rather than specialize in one. Flexible strategies predispose toward generalist, rather than specialist phenotypes that can be strategically deployed across a wide range of environments. As with biological preparedness, developmental plasticity and behavioral flexibility are also ultimately genetically influenced, and subject to the selective pressures of genetic accommodation (including assimilation) of environmentally-triggered phenotypic changes during development. For easy reference, a Glossary (see Supplementary Material) of technical terms that have been used throughout this paper has been assembled.

In terms of SD-IE theory, those individuals that are higher on strategic *differentiation* effort (who exhibit *slower* life history and are higher-K strategists) are likely to be more phenotypically plastic when compared with those higher on strategic *integration* effort (who exhibit *faster* life history and are lower-K strategists), who should be more phenotypically flexible. Those higher on K should also be less phenotypically prepared compared with those lower on K, who exhibit faster maturational rates and receive relatively much less parental and nepotistic investment. Low-K individuals must therefore be prepared for high flexibility early in life with respect to tactics that can be differentially deployed contingent upon the immediate environment encountered. Levels of phenotypic preparedness might be less consistent among high-K populations where selection is more uniformly directional with respect to the occupation of specific micro-niches, rather than diversifying with respect to diverse micro-niches. Under those more stable conditions, preparedness is expected to be more heritable and higher, although this will be traded off against a lower amount of shorter-term flexibility, allowing individuals to pass on highly genetically assimilated social roles across generations. This might be interpreted as the ongoing evolution in humans of what are comparable in some ways to eusocial insect polyethisms (behaviorally specialized “castes”), which also appear to be controlled by a combination of genetic and epigenetic influences (Cahan et al., 2010; Lo et al., 2010; Patalano et al., 2012).

SD-IE among humans has been corroborated with many latent and outcome variables comprising the life history continuum: these include the behavioral and cognitive indicators in the Arizona Life History Battery (ALHB; collectively measuring the *K*-Factor), various personality dimensions, measures of physical and mental health (Figueredo et al., 2013b), and at the group-differences level, fertility, longevity, infant mortality, cranial capacity, crime rate indicators, economic indicators, overall life satisfaction, prevalence of sexually transmitted diseases, skin reflectance, divorce rate, and height (Fernandes and Woodley, 2013; Armstrong et al., 2014; Woodley and Fernandes, 2014; Woodley et al., 2014). However, the genetic characteristics and consequences of SD-IE have not yet been explored, which leaves various important questions unanswered: For example, is the SD-IE effect heritable?

### Predictions

On the basis of the aforementioned open research question, four testable predictions have been formulated which will be explored using two large and demographically representative samples of twins sourced from the US and Sweden:

Prediction 1: Individual differences in the degree of biological LH preparedness (Figueredo et al., 2006), i.e., the degree to which a population might be predisposed (or prepared) toward developing a particular specialized set of life history traits, are partly heritable. Individual differences in biological preparedness may have been shaped by a variety of related evolutionary pressures, such as those relating to genetic accommodation (West-Eberhard, 2003), and also biased epigenetic rules of development, which may stem from gene-culture co-evolution (Lumsden and Wilson, 1983).

Prediction 2: The individual differences-level SD-IE effects themselves, which as in previous work, are defined as the effect of the level of the higher-order life history factor upon its loadings on the lower-order factors, will be heritable, evidencing the principle of genetic constraints upon phenotypic plasticity (Waddington, 1953; Lumsden and Wilson, 1983; West-Eberhard, 2003). Estimating the heritability of SD-IE is equivalent to estimating the heritability of the aforementioned continuum of plasticity vs. flexibility.

Prediction 3: The higher-order life history factor (Super-K), as well as its lower-order factors, will be less heritable among slower than among faster LH strategists. This follows on from the observation made in previous work that the SD-IE effect appears to be most pronounced on measures of life history that are more strongly correlated with K than those that are less so (Figueredo et al., 2013b). Put more simply, when a LH component disaggregates at higher levels of the Super-K factor (i.e., when strategic differentiation occurs), the effect is biggest on those components that load more strongly on the heritable latent factors. This weakening of these correlations should therefore attenuate the heritability of LH among those who are most strategically differentiated, i.e., those highest on the latent factors.

Prediction 4: It has been demonstrated that weaker intergenerational mobility prevails in Sweden compared to North-American countries in terms of skills and expertise (Western and Wright, 1994), and that Swedes exhibit relatively high status persistence across surname generations (Clark et al., 2013). Therefore the Swedish sample should exhibit higher levels of heritability across traits relative to the American sample, evidencing a greater degree of assimilation of individuated life history strategies.

## Methods

### Samples of participants

*Sample 1* was comprised of a nationally-representative subsample of 316 dyads of monozygotic (MZ) twins and 274 dyads of same-sex dizygotic (DZ) twins (ages 25–74) from Wave 1 (1995–1996) of the Survey of Midlife Development in the United States (MIDUS; Brim et al., 2000), on which previous life history (LH) analyses had been performed (Figueredo et al., 2004, 2007; Figueredo and Rushton, 2009). The MIDUS Survey consisted of a telephone interview and two follow-up mail surveys given to a nationally representative sample, collected in two longitudinal data collection waves, the first wave over a one year period from 1995–1996 (*n* = 7108), and the second wave over a 2 year period from 2004–2006 (*n* = 4963). This sample was limited to English speakers in the United States between the ages of 25–74 (at Wave 1) and 35–86 (at Wave 2), and contained data on singletons (non-twins) as well as on a genetically informative sample of same-sex MZ and DZ twins pairs. The MIDUS data were used with written permission from the MIDUS program, obtained through their web site (http://www.midus.wisc.edu/). The use of this archival data was also approved by the Institutional Review Board of the University of Arizona, Office for the Responsible Conduct of Research, Human Subjects Protection Program (http://orcr.arizona.edu/hspp).

*Sample 2* was comprised of a sample of Swedish Twins, on which a similar selection of LH-related variables were available. The data were collected from a large sample of twins from the Swedish Twins Registry (STR)—the STAGE cohort (Lichtenstein et al., 2002) with approximately 32,000 twins born between 1959 and 1985. Zygosity was determined by questions about intra-pair similarities and was subsequently confirmed in 27% of the twins in the STR using genotyping. For further details on the STAGE cohort and zygosity determination in the STR see Lichtenstein et al. (2002, 2006). Data collection was conducted via web-survey. An invitation was sent via surface mail to ~32,000 twins, 11,543 of whom completed at least one instrument in a web questionnaire via the Internet. The final sample used in the present study, after multiple imputation for missing data, contained 863 MZ and 475 same-sex DZ twin pairs. Use of this sample was approved by the Regional Ethics Review Board in Stockholm. Each participant provided written consent.

### Measures

The measures used in the analyses involving the MIDUS cohorts (Covitality, the K factor and the GFP) have been detailed in other publications (Figueredo et al., 2004, 2007; Figueredo and Rushton, 2009). The measures employed in the analyses involving the Swedish STAGE data were not precisely equivalent to those employed in the MIDUS based study. As a measure of *K*, the *Mini K* was employed (Figueredo et al., 2006). This is a 20 item short-form measure of *K* which exhibits adequate reliability (0.73) and an excellent validity (0.91) (Figueredo et al., 2014). A 44-item short version of the Big five Inventory (John et al., 1991) was employed as a measure of the GFP (the BFI-44). For Covitality, two questions tapping the domain of mental health were employed, one of which measured depression (Magnusson Hanson et al., 2014), with the second measuring emotional exhaustion (Magnusson Hanson et al., 2008). These were reverse scored such that high mental health equated to low depression and emotional exhaustion.

### Missing data imputation

Missing data must be addressed when utilizing longitudinal data. If large amounts of missingness are present in a dataset, this might bias parameter estimates and *p*-values, increasing type 1 or type 2 error (Schlomer et al., 2010). For the analyses, missing data were handled with *multivariate imputation* (Gorsuch, 1983; Figueredo et al., 2000) and *multiple imputation*, using PROC MI in SAS. Thus, data for each twin with in each category of zygosity were imputed as a separate individual prior to disaggregating and then recombining them for further analysis. Twins were then *matched* within twin dyads, and later randomly *paired* with opposite-zygosity twin dyads for heritability analyses, as described below. To avoid confusion, the use of the expression “matching” *within* each twin dyad will be restricted to mean the linking of each twin with each corresponding co-twin (identical sibling). Instead the expression “pairing” *between* these twin dyads is used, specifically meaning the random assignment of DZ twin dyads to MZ twin dyads not naturally associated across zygosities.

Multivariate imputation (MVI) involved estimating unit-weighted factor scores for the component scales and composite lower-order factors using: (1) the means of the standardized scores for all items that were not missing within each scale and (2) the means obtained from the standardized scores for all the indicator scales not missing within each factor (Figueredo et al., 2000). Most of the scale and lower-order factor scores were recovered this way. Unit-weighted factor scoring was applied, in order to avoid problems associated with the sample-specificity of factor scoring coefficients produced by standard errors of inconsistent magnitudes across different samples (Gorsuch, 1983). These unit-weighted factor structures are therefore simply part-whole correlations (here termed “unit-weighted factor loadings” for convenience) between the latent composites and each of their component indicator measures. Although some of the samples were of sufficient size to reliably estimate differentially-weighted factor scores and factor loadings, this one method was applied throughout for consistency.

For those missing data that remained on the scale and factor scores, the EM algorithm, as implemented by SAS PROC MI was employed. Each of the twin datasets were assigned 30 multiple imputations using this procedure. These were then aggregated at the lower-order factor level across the 30 “multiple imputations” (using SAS PROC MEANS).

### Data aggregation and analytic procedures

The Continuous Parameter Estimation Method (CPEM; Gorsuch, 2005) was employed. This permits the change in the covariance between two variables (such as a higher-order multivariate construct like *Super-K* and a constituent lower-order latent variable like *Covitality*) to be determined throughout the full range of another variable (such as the overall level of *Super-K*). For all LH domains sampled, correlation coefficients were estimated at the individual level by taking the cross-product of the standardized (*Z*) scores of each individual's performance on the relevant subscales.

Pearson's Product-Moment Correlation Coefficient is defined as the mean cross-product of the standardized (“*Z*”) scores:

(1) $$\Sigma (Zx^{*}Zy)/N$$

It follows that the group mean of these individual-level cross-products automatically becomes the correlation coefficient for each group under consideration. This is mathematically inevitable by definition. Therefore, the cross-product itself (*Zx* ^*^ *Zy*) can be used as the individual-level “raw score” in CPEM to estimate the varying amount of strategic integration or differentiation “effort” in each group. Thus, computing and comparing the group means of these cross-products using ANOVA automatically calculates and compares group-level Pearson Correlation Coefficients. This tests the degree to which the strength of this relationship varies between any discreet groups.

When using more traditional methods for identifying changes in the strength of the correlation coefficient between groups, it is necessary to acquire samples of at least 75–100 respondents in each group, so as to stabilize the correlation coefficients for comparison. As a graded method, CPEM does not require the polytomization of continuous distribution, by potentially problematic methods such as the median split (Cohen and Cohen, 1983; MacCallum et al., 2002). Furthermore, CPEM permits one to regress the individual cross-products of the z-scores on continuous as well as categorical predictor variables, facilitating the application of this method to multiple regression/correlation (MRC) analyses as well as ANOVAs.

In the present study, Continuous Parameter Estimates (CPEs) for all *individual*-level parameters were obtained by means of simple cross-multiplications of the standardized scores involved in each model parameter. For example, SD-IE effects were estimated by cross-multiplying the standardized unit-weighted higher-order factor scores with the standardized unit-weighted factor loadings on all three lower-order factors. The mean values of these individual-level CPEs automatically yield Pearson's Product-Moment Correlation Coefficients in the group-level aggregate SD-IE effects.

To be able to estimate *dyadic*-level parameters using CPEM, 50 randomly-assigned pairings of DZ twin dyads for each of the MZ twin dyads were performed. This procedure produced on average 45 usable pairings (ranging from 39 to 50, due to differing numbers of MZ and DZ twins along with the vagaries of the random assignment process) for each MZ twin dyad, which were then then aggregated across these randomly-assigned pairings to produce the final CPEs for each MZ twin dyad, each representing a *single genome*, with which a unique heritability coefficient can be associated. The dyadic-level parameter estimates, such as heritability coefficients, therefore apply to each MZ twin dyad, and cannot be uniquely associated with any particular DZ twin dyad, because the latter differ systematically in their genotypes within matched dyads whereas MZ twins do not.

As described above, first, the CPEs of SD-IE parameters for each individual co-twin were calculated, regardless of zygosity. After *matching* and then *pairing* (as per the restricted definitions given above) twin dyads, heritability coefficients for each randomly-assigned MZ-DZ dyad pairing were estimated, and then aggregated across these pairings to obtain “individual-genome-level” MZ dyadic means, for the SDIE effects. Individual-genome level heritabilities were estimated by CPEM by applying the Falconer Formula (Figueredo et al., 2004) separately to each random MZ-DZ dyad pairing:

(2) $$h^{2}=2(r_{MZ}-r_{DZ})$$

The mean values of these individual-genome-level CPEs of heritability coefficients automatically yield values approximating the group-level aggregates obtained using conventional methods (Figueredo et al., 2004; Figueredo and Rushton, 2009).

Although more sophisticated analytical techniques are now available for the estimation of heritability coefficients, such as biometric structural equations models (SEM; Rijsdijk and Sham, 2002), and while these more advanced methods are capable of providing additional information such as decomposing the shared genetic variance into additive and non additive components, the simpler but older Falconer Formula is more effective when using CPEM. As there is no obvious method of obtaining the needed parameter estimates from SEM, the whole point of the present analytical strategy is to develop a method to generate individual-genome-level heritability coefficients, which are here termed individual *transmissibilities* on the basis that they capture the degree to which a given trait is reliably transmitted across generations (as reflected in the degree of twin-genomic similarity). Furthermore, this procedure entailed 50–100 random pairings of DZ twin dyads with MZ dyads, for a combined cross-cultural sample of over 1000 MZ dyads. The estimation of these model parameters by SEM would have required 5,000–10,000 such models to be estimated and then meta-analytically aggregated. Such an approach would have been unwieldy, and difficult to interpret, as well as too computationally intensive for currently available equipment. Finally, the aggregate-level heritabilities of the higher- and lower-order factors in the MIDUS data have already been analyzed by means of both the Falconer Formula (Figueredo et al., 2004) and a biometric Common Pathway Model SEM (Figueredo and Rushton, 2009), yielding nearly identical results for broad-sense heritabilities. Of course, the latter method was able to decompose the heritability coefficients into additive and non additive components, which, as acknowledged above is its major theoretical advantage, in addition to yielding narrow-sense heritability coefficients that were (as expected) lower than the broad-sense heritability coefficients, which were the ones necessarily obtained by the old Falconer method. This was the basic reason why the MIDUS data were reanalyzed with a Common Pathway Model SEM (Figueredo and Rushton, 2009), as the goal of that study was to estimate the non additive genetic variance in supporting the evolutionary-genetic hypothesis of recent and directional selection for these traits in human populations.

In double checking these relatively novel CPEM procedures, the group-level aggregations in an alternative sequence were performed in order to demonstrate that approximately the same SD-IE effect parameter estimates were recoverable within an acceptable margin of rounding error. These alternative SD-IE estimates were obtained by means of bivariate regressions, specified as follows: (1) using the standardized higher-order common factor *scores* as the single predictor variable; and (2) using the standardized factor *loadings* of the higher-order common factor on each of the lower-order factors as the criterion variables. This is how SD-IE effects have been estimated previously (Figueredo et al., 2013b). It is necessary to make sure that these new CPEM procedures produced the same results as the old one. To examine the robustness of the novel procedure as compared with the conventional one, these bivariate regressions were performed separately on each of the randomized MZ-DZ dyad pairings and then aggregated across them as literal replications, simulating what would have been obtained when meta-analyzing the regression results of 50 independent samples of the same population to obtain a single, synthetic population-level regression coefficient. The results of this alternative sequence of data aggregation and analysis produced almost identical parameter estimates regardless of which method was used. These complementary results can be made available upon request.

After these basic CPEM parameters were estimated, the two samples (Sweden and USA) were compared by simple one-way analyses of variance and tests for equality of means and variances were conducted. This was done first for the following: (1) the heritabilities of the higher-order and lower-order life history factors, (2) the heritabilities of the factor loadings of the lower-order life history factors, (3) and the heritabilities of the SD-IE effects of the higher-order factor upon the factor loadings of the lower-order life history factors. Secondly, this same test was repeated for the following derived parameter estimates: (4) the correlations among the Super-K factor and the heritabilities of the higher-order and lower-order life history factors, (5) the correlations among the Super-K factor and the heritabilities of the factor loadings of the lower-order life history factors, and (6) the correlations among the Super-K factor and the heritabilities of the SD-IE effects of the higher-order factor upon the factor loadings of the lower-order life history factors.

Finally, variable skewness has been consistently demonstrated in previous SD-IE tests that used CPEM to have negligible effects on the estimates of SD-IE effect magnitudes (Fernandes and Woodley, 2013; Figueredo et al., 2013b; Armstrong et al., 2014; Woodley and Fernandes, 2014; Woodley et al., 2014). Therefore this has not been controlled in the present study.

## Results

Table 1 presents the results of the tests for SD-IE, that is, the effect of the level of the Super-K upon its loadings on the lower-order factors, among monozygotic twins. SD-IE effects were highly comparable (not statistically different from each other at an *a priori* significance level of *p* < 0.05) between the two countries. The SD-IE parameters of the United States sample have already been reported in a previous paper (Figueredo et al., 2013b), but have not before been systematically compared to a cross-cultural comparison group. Levene's Test for Equality of Variances was used to evaluate the null hypothesis that the variances in two samples are statistically equivalent (Levene, 1960).

**Table 1:** **Correlations between Super-K and lower-order factors (SD-IE Effects) of randomly assigned monozygotic twins**.

**Variable**	**American sample**		**Swedish sample**		**Equality of Variances *F*(1, 1177)**	***P***	**Equality of means *F*(1, 1177)**	***P***
	***r* (*SE*)**	***P***	***r* (*SE*)**	***P***				
**RANDOM MZ TWIN 1**								
*K*-factor	−0.32 (0.14)	<0.001	−0.33 (0.09)	<0.001	0.02	0.88	0.02	0.88
GFP	−0.22 (0.14)	<0.001	−0.33 (0.09)	<0.001	0.11	0.74	0.35	0.55
Covitality	−0.40 (0.16)	<0.001	−0.41 (0.09)	<0.001	0.00	0.99	0.01	0.93
**RANDOM MZ TWIN 2**								
*K*-factor	−0.37 (0.13)	<0.001	−0.32 (0.09)	<0.001	0.01	0.93	0.08	0.78
GFP	−0.31 (0.14)	<0.001	−0.31 (0.09)	<0.001	0.09	0.77	<0.01	0.99
Covitality	−0.42 (0.14)	<0.001	−0.45 (0.10)	<0.001	0.02	0.88	0.04	0.84

Table 2 presents the results of the heritability analysis involving Super-K and its components, the factor loadings of the components and also the value for the SD-IE effects associated with each lower-order factor. This systematic comparison also permitted determination of whether the values estimated for the Swedish twins were higher than those of the US sample, which would, consistent with predictions, suggest that the Swedes have been evolving toward greater degrees of genetic stratification. It should be noted that, although the differences in heritability between countries were not significant, the Swedish sample nonetheless presented a trend toward higher average heritabilities for factor loadings (0.56 ± 0.22, compared to 0.37 ± 0.20 in the American sample) and for SD-IE (0.57 ± 0.40, compared to 0.42 ± 0.26 in the American sample), while mean heritabilities for the LH factors were quite similar (0.50 ± 0.06 in the Swedish compared to 0.51 ± 0.11 in the American sample).

**Table 2:** **Heritabilities of higher-order and lower-order life history factors, higher-order factor loadings on lower-order life history factors, and SD-IE effects on lower-order life history factors**.

**Variable**	***h^2^* (*SE*)**				**Equality of variance *F*(1, 1177)**	***P***	**Equality of means *F*(1, 1177)**	***P***
	**American sample (*N* = 316)**	***P***	**Swedish Sample (*N* = 863)**	***P***				
**FACTORS**								
Super-K	0.58 (0.14)	<0.001	0.57 (0.09)	<0.001	0.31	0.58	<0.01	0.97
*K*-Factor	0.53 (0.12)	<0.001	0.37 (0.08)	<0.001	0.64	0.42	1.13	0.29
GFP	0.52 (0.12)	<0.001	0.67 (0.08)	<0.001	0.46	0.50	0.98	0.32
Covitality	0.48 (0.19)	<0.001	0.47 (0.10)	<0.001	0.55	0.46	<0.01	0.99
**FACTOR LOADINGS**								
*K*-factor	0.40 (0.18)	<0.001	0.62 (0.23)	<0.001	0.53	0.47	0.32	0.57
GFP	0.14 (0.13)	<0.001	0.62 (0.21)	<0.001	1.27	0.26	1.88	0.17
Covitality	0.57 (0.37)	<0.001	0.41 (0.27)	<0.001	0.18	0.67	0.08	0.78
**SD-IE**								
*K*-factor	0.30 (0.22)	<0.001	0.55 (0.43)	<0.001	1.25	0.26	0.14	0.71
GFP	0.30 (0.15)	<0.001	0.61 (0.40)	<0.001	2.05	0.15	0.24	0.63
Covitality	0.64 (0.44)	<0.001	0.49 (0.41)	<0.001	0.02	0.88	0.03	0.87

Table 3 presents the correlations between the level of each variable and the individual-level heritability estimates, or individual *transmissibilities*, associated with each variable. Negative correlations indicate that as the indicator-level increases, the level of heritability decreases. Correlations were comparable between the two samples for all traits.

**Table 3:** **Correlations between the higher-order life history factor and the heritabilities of the Super-K Factor, the heritabilities of the higher-order factor loadings on lower-order life history factors, and the heritabilities of the SD-IE effects on lower-order life history factors**.

**Variable**	**Pearson's *r* (*SE*)**				**Equality of variances *F*(1, 1177)**	***P***	**Equality of means *F*(1, 1177)**	***P***
	**American sample**	***P***	**Swedish sample**	***P***				
***H^2^* OF FACTORS**								
Super-K	−0.31 (0.12)	<0.001	−0.29 (0.09)	<0.001	0.03	0.85	0.02	0.90
*K*-factor	−0.17 (0.09)	0.002	−0.23 (0.07)	<0.001	0.03	0.86	0.26	0.61
GFP	−0.17 (0.07)	0.002	−0.15 (0.07)	<0.001	0.11	0.75	0.02	0.89
Covitality	−0.34 (0.14)	<0.001	−0.25 (0.08)	<0.001	0.47	0.49	0.33	0.57
***H^2^* OF FACTOR LOADINGS**								
*K*-factor	−0.23 (0.15)	<0.001	−0.27 (0.13)	<0.001	0.53	0.76	0.02	0.89
GFP	−0.20 (0.14)	<0.001	−0.24 (0.13)	<0.001	0.05	0.82	0.04	0.85
Covitality	−0.31 (0.16)	<0.001	−0.26 (0.12)	<0.001	0.17	0.68	0.05	0.82
***H^2^* OF SD-IE**								
*K*-factor	−0.21 (0.17)	<0.001	−0.21 (0.14)	<0.001	0.06	0.81	<0.01	0.99
GFP	−0.18 (0.17)	0.001	−0.20 (0.14)	<0.001	0.02	0.89	0.01	0.93
Covitality	−0.26 (0.17)	<0.001	−0.23 (0.14)	<0.001	0.05	0.82	0.02	0.90

## Discussion

This study expands on previous examinations of the heritability of the higher-order Super-K factor and its lower-order factors (K-Factor, GFP, and Covitality) in several ways. Firstly, a method for estimating the heritability of factor loadings and the heritability of interindividual variations in factor loadings (i.e., the heritability of SD-IE) was proposed based on CPEM. Secondly, this represents the first attempt to estimate the heritability of some of these higher-order factors in populations other than North-Americans (the heritability of the Mini-K in Sweden has been established in a previous publication, using conventional biometric SEM; Woodley of Menie and Madison, 2015). It was demonstrated that the heritability coefficients of LH factors, of their factor loadings, and of their SD-IE effects are statistically equivalent in Sweden and in the United States. Finally, this method enabled the testing of four specific predictions set out in the introduction: (1) that individual-level factor loadings of the higher-order life history factor on its lower-order factors will be partially heritable, evidencing some degree of genetic preparedness; (2) that the individual-level SD-IE effects themselves (the effects of the level of the higher-order life history factor upon its loadings on the lower-order factors) will be heritable, evidencing genetic influences upon individual differences in *flexibility* vs. *plasticity*; (3) that the higher-order life history factor (Super-K), as well as its lower-order factors, will be less heritable at its higher levels than at its lower-levels; (4) that the Swedish sample will exhibit higher levels of heritability across traits relative to the American sample, evidencing a transition toward greater preparedness and lower plasticity in the evolution of social polyethism.

Prediction 1 was validated in the current study, with the factor loadings in both the US and Swedish samples indicating modest to high levels of heritability (the lowest was in the case of the GFP from the US sample, *h*^2^ = 0.14, all other estimates were >0.40). Consistent with prediction 2, the SD-IE effects were also heritable in both samples (with *h*^2^ ranging from 0.30 to 0.64). This indicates that the degree to which individuals are predisposed toward the canalization of a particular phenotype by virtue of the strength of their individual factor loadings is partly under genetic control. Furthermore, the degree to which individuals are capable of specialization once a particular developmental trajectory has been assumed is also partly under genetic control.

Prediction 3 was validated, as in both the Swedish and US samples the heritability of Super-K was observed to significantly decrease as a function of increasing levels of the latent variable. This is consistent with the observation that across scales, the SD-IE effect is concentrated on the strongest measures of K, thus the decline in heritability likely results from the fact that the K-factor itself weakens at high levels. The systematic comparisons revealed no significant differences between the samples in terms of the magnitudes of these effects across the two populations.

Concerning Prediction 4, the average heritability of the measured traits was not significantly higher in the Swedish sample than in the United States sample. Prediction 4 was therefore not supported, although the pattern of results suggested that the Swedish mean heritabilities might have been somewhat higher given sufficient statistical power or lower inter-individual variance in heritability coefficients. This fails to support the notion that the Swedish population is relatively more genetically differentiated in life history allocations than the North American one.

It must be noted that in Sweden the *K* factor was assessed with the Mini-K, therefore its heritability was expected to be lower than that of the American *K* factor (which was measured with the full ALHB) due to the fact that weaker measure reliabilities tend to attenuate correlations (Hunter and Schmidt, 2004). This proved to be the case, although the difference was non significant.

Although the ability to estimate individual-genome transmissibilities might seem like a purely academic point of statistical legerdemain, it opens up wide-ranging possibilities for future re studies. In this study, it was found that the mean levels of the higher-order “Super-K” life history factor for each MZ Twin Dyad predicted a variety of SD-IE effects, as well as the transmissibilities of their higher-order and the lower order factor scores, the factor loadings of the lower-order indicators, and the genomic transmissibilities of all of these parameters. Considering these analyses a “proof of concept” as well as an interesting finding in their own right, this same logic can be extended to model the covariation of transmissibility coefficients with any other set of predictor or criterion variables. For example, other causal influences, whether genetic or environmental in origin, could be used to investigate potential moderation effects on these transmissibilities.

Thus, the ability to estimate individual-genome transmissibilities opens up wide-ranging possibilities for future studies. For example gene-gene and regulatory gene interactions with other adaptive traits might be involved in the development of: (1) preparedness or plasticity in behavior, at the developmental level; and (2) the extrinsic variability that might be present in the external environment, may provide the selective pressures over evolutionary time for these conditional adaptations. Molecular biomarkers could in principle be linked to individual transmissibilities, therefore the epigenetic biochemical mechanisms of preparedness and plasticity could be elucidated at the proximate level of causation.

These exciting possibilities were opened up primarily by the application of the Continuous Parameter Estimation Model (CPEM) to heritability analysis, which has never been attempted hitherto. Furthermore, one of the advantages of CPEM is that the CPEs for these individual transmissibilities can be subjected to subsequent analyses by a wide array of other parametric statistical methods. These include analysis of variance and multiple regression (as illustrated in the present paper), but may also be extended to path analysis, factor analysis, hierarchical linear models, generalizability theory analyses, etc. This is not an entirely different method for constructing biometric models, merely an addition to the armamentarium of complementary analytical tactics that may be applied.

### Conflict of interest statement

The authors declare that the research was conducted in the absence of any commercial or financial relationships that could be construed as a potential conflict of interest.
