# Supplementary material for: Strategic differentiation and integration of genomic-level heritabilities facilitate individual differences in preparedness and plasticity of human life history
Source: Front Psychol. 2015 Apr 22;6:422. doi: 10.3389/fpsyg.2015.00422 (PMC4405998; doi:10.3389/fpsyg.2015.00422)
Supplement: Supplementary file 1 [file DataSheet1.DOCX]

**Appendix A – Glossary**

**Behavioral Flexibility:** The evolved ability to make short-term epigenetic modifications to biologically prepared associations in response to the differing adaptive problems posed by transient situations. This property is distinct from long-term or permanent epigenetic modifications in response to adaptive problems posed by environmental circumstances that are relatively stable over developmental time, which are typically considered cases of phenotypic plasticity rather than flexibility. The changes wrought by the mechanisms of phenotypic plasticity are often irreversible, whereas those produced by behavioral flexibility are not.

**Biological Preparedness**: The initial strength or starting potency of an evolved association between an environmental stimulus, or *cue*, and an organismic response, or *means*, as optimized by selection to reflect the mean ecological validity coefficient of that relation over evolutionary time (Figueredo et al. 2006) . This parameter is independent of the phenotypic plasticity of that association.

**Broad-Sense Heritability**: The totality of the genetic contributions to trait variance. It includes both additive and nonadditive sources of genetic variance, which would include dominant and epistatic genes.

**Carrying Capacity**: The maximum population size of a species or population that a particular environment can support indefinitely, given the resources available in that environment (Hui 2006).

**Character Displacement**: The accentuation of differences among species or populations whose distributions overlap geographically, as a consequence of competition between the species or groups for limited resources (Brown and Wilson 1956).

**Continuous Parameter Estimation Model**: A statistical model that permits the calculation of case-level (as opposed to sample-level) correlations, variances, and other parameters traditionally considered discrete (meaning applicable only at the aggregate level; Gorsuch 2005), allowing such truly continuous parameters to be used in correlative, regressive, and other types of analyses.

**Covitality**: Although this term was originally used to refer to the positive correlations among any set of traits perceived as indicative of higher genetic quality, and as such referred simply to the opposite of “comorbidity” (Weiss et al. 2002), it has been coopted to refer to the common factor (at Stratum II of the life history strategy hierarchy) comprising mental and physical health traits in general (Figueredo et al. 2004).

**Differentially-Weighted Factor:** A latent variable (i.e. common factor) estimated from the factor scoring coefficients produced by a factor analysis for its various indicators, which are ultimately based on the specific magnitude of the factor loading of each indicator. Indicators that share more variance with others are given larger weights towards the estimation of the common factor.

**Epigenetic Rules of Development:** The system of gene–by–environment interactions that guide the development and expression of phenotypic traits. Epigenetic rules may vary from population to population, allowing the evocation of different traits to different degrees based on the local adaptive optima. Examples of how altered epigenetic rules of development may change the pattern of traits within a population can be found in the form of increased mating effort and fertility spikes among women accompanying increased experiential exposure to levels of extrinsic morbidity and mortality, stemming from an increase in environmental harshness and unpredictability (e.g. after a terrorist attack or natural disaster; e.g. Rodgers et al. 2005).

**Gene-Culture Co-Evolution**: The dynamic interaction between changes in selection pressures stemming from changes in culture and corresponding adaptive changes in gene frequencies (Cavalli-Sforza and Feldman 1981).

**Gene-Gene Interactions**: Where the product of one gene or set of polygenes upregulates or downregulates the expression of another gene or set of polygenes. Also known as epistasis.

**General Factor of Personality**: The moderately to highly heritable common factor variance (at Stratum II of the life history strategy hierarchy) latent among the various traits identified in human personality research, regardless of the specific assessment inventory used. It captures a dimension that has been theorized as manifested by high social effectiveness and altruism at one pole, and low social effectiveness, shading into personality disorders at the other (Rushton et al. 2008).

**Genetic Accommodation:** Gene frequency change due to selection on the genetically variable polygenic regulation of a novel trait produced by phenotypic plasticity in response to an environmental change (West-Eberhard 2003)

**Genetic Assimilation:** A special case of genetic accommodation, applying only to environmentally-initiated traits that are under positive selection pressure. This selection is on the genetic regulation of phenotypically plastic traits, and has the effect of increasing the degree of the genetic influence upon them (Waddington 1953; West-Eberhard 2003)

**Genetic Constraints:** Patterns of genetic variation or covariation that that reduce or prevent the response to natural selection, such as epistasis and pleiotropic trade-offs, or additive genetic variance and covariance. These constraints may represent a product of Genetic Accomodation/Assimilation.

**Genomic Transmissibility:** The genomic-level heritability of traits, as derived from a comparison of individual monozygotic twin-pairs to a random subsample of dizygotic twin-pairs.

**Higher-Order and Lower-Order Factors:** A Higher-Order Factor is a latent variable (i.e. a common factor), as indicated by two or more latent variables which are substantially correlated, representing a higher level of data aggregation. A Lower-Order Factor is one among these substantially correlated latent variables that give rise to a Higher-Order common factor by virtue of the variance they share. These two terms are employed when there are at least two levels of trait latency in a field or study, to differentiate between levels of data aggregation and abstraction.

**K-Factor:** The moderately to highly heritable common factor (at Stratum II of the life history strategy hierarchy) among cognitive and behavioral indicators of traits that represent slow life history in humans, including long-term pair-bonding, long-term planning, the capacity for delay of gratification, parental investment, nepotism towards extended kin, altruism towards the community, and sexual restraint. The psychometric approach to assessing the K-Factor relies on converging self-reported measures of psychosocial indicators of these slow life history traits, and as such assesses the *process* of resource allocation into different components of fitness, that is, it taps the bioenergetic adaptations being actually executed.

**Levene’s Test for Equality of Variances:** A statistic which tests the null hypothesis that the variances in two samples are equal (Levene 1960). Higher scores in this statistic reflect bigger difference between the variances of the samples

**Measurement Reliability:** The proportion of replicable variance in a measure, as adjusted for the degree of random error resulting from any imprecisions in the measurement process.

**Multiple Imputation:** A simulation-based approach to the handling of missing data, which relies on replacing each missing datum with more that one simulated value, producing multiple reconstructed versions of the complete data (Schafer 1997). Among the currently most popular of these methods is the EM algorithm, which is implemented in several major software packages such as SAS, SPSS, and HLM (Figueredo et al. 2000).

**Multivariate Imputation:** The application of unit-weighted factor scoring to impute the latent variable scores from the available subset of indicators, when incomplete but deemed sufficient for a valid estimate, without imputing the score of the missing indicator(s) themselves. This is a form of “within-subjects” imputation that does not rely on using scores from other individuals but produces an imputed value solely from the data available for every separate case (Figueredo et al. 2000).

**Narrow-Sense Heritability:** The genetic variance associated with individual differences in a trait that is due purely to additive genes. This includes genes that individually have small effects, but cumulatively account for large amounts of variance (i.e. polygenes).

**Ontogeny:** The history of an organism’s phenotypic modifications across its life span.

**Phenotypic Plasticity:** The range over which the strength of a prepared association may be adjusted over developmental time by evolved epigenetic mechanisms, based on the experience of the individual organism with that association, as localized by the specific time and space inhabited, as optimized by selection to reflect the *variance* of the ecological validity coefficient of that relation over evolutionary time (Figueredo et al. 2006). This parameter is independent of the biological preparedness of the association.

**Polytomization:** Division of a sample or dataset into subgroups, based on arbitrary cutoff points in the distribution of a trait. A well-known example is the median split, which gives rise to two groups (i.e. dichotomization).

**Regulatory Genes:** These genes regulate the behavior of other genes hierarchically, either via gene–by–environment or gene–by–gene interactions. These genes play an important role in controlling the timing and expression of complex, adaptive traits such as in the development of a coherent and coordinated life history strategy.

**Social Polyethism:** The differentiation of social behaviors that may occur in response to complex ecological contexts, and is in turn generative of even more social complexity.

**Social Selection:** An evolutionary process that occurs as a result of increases or decreases in fitness resulting from interactions between individuals within social contexts.

**Socio-Ecological Micro-Niche:** The product of all of the social interactions that define specific modes of resource extraction within a social context (e.g. specialized occupations in human societies practicing division of labor).

**Strategic Differentiation:** The effort allocated by those with slow life histories into the development of specialized profiles of physiological, psychosocial, and cognitive traits in response to both heredity and gene–by–environment interactions, which serve to calibrate the pattern of canalization (meaning the shaping of a developmentally plastic phenotype by genetic accommodation/assimilation). Such specialist phenotypes are selected for the adaptive function of managing heightened competition in social species given stable and densely populated ecological contexts.

**Strategic Integration:** The effort allocated by those with fast life histories into the homogenization of the development of physiological, psychosocial and cognitive traits. This gives rise to a “Jack-of-all-trades” pattern of life history traits, which is selected for the adaptive function of contingency management given the unpredictable ecological contexts favoring fast life history.

**Super-K Factor:** The moderately to highly heritable higher-order (Stratum III) common factor underlying the shared variance among the K-Factor, the Covitality Factor, and the General Factor of Personality, representing the common genetic and epigenetic influences upon health, personality, and life history strategy (Figueredo et al. 2004).

**References**

Brown, W. L., and Wilson, E. O. (1956). Character displacement. Syst. Zool. 5, 49–65. doi: 10.2307/2411924

Cavalli-Sforza, L. L., and Feldman, M. W. (1981). Cultural Transmission and Evolution: A Quantitative Approach. Princeton, NJ: Princeton University Press.

Figueredo, A. J., Hammond, K. R., and McKiernan, E. C. (2006). A Brunswikian evolutionary developmental theory of preparedness and plasticity. Intelligence 34, 211–227. doi: 10.1016/j.intell.2005.03.006

Figueredo, A. J., McKnight, P. E., McKnight, K. M., and Sidani, S. (2000). Multivariate modeling of missing data within and across assessment waves. Addiction 95, 361–380. doi: 10.1046/j.1360-0443.95.11s3.6.x

Figueredo, A. J., Vásquez, G., Brumbach, B. H., and Schneider, S. M. (2004). The heritability of life history strategy: the k−factor, covitality, and personality. Biodemogr. Soc. Biol. 51, 121–143. doi: 10.1080/19485565.2004.9989090

Figueredo, A. J., Wolf, P. S., Olderbak, S. G., Gladden, P. R., Fernandes, H. B. F., Wenner, C., et al. (2014). The psychometric assessment of human life history strategy: a meta-analytic construct validation. Evol. Behav. Sci. 8, 148–185. doi: 10.1037/h0099837

Gorsuch, R. L. (2005). Continuous parameter estimation model: Expanding the standard statistical paradigm. J. Sci. Fac. Chiang Mai Univ. 32, 11–27.

Hui, C. (2006). Carrying capacity, population equilibrium, and environment’s maximal load. Ecol. Model. 192, 317–320. doi: 10.1016/j.ecolmodel.2005.07.001

Levene, H. (1960). “Robust tests for equality of variances,” in Contributions to Probability and Statistics, eds I. Olkin, S. G. Ghurye, W. Hoeffding, W.G. Madow, and H. B. Mann (Stanford, CA: Stanford University Press), 278–292.

Rodgers, J. L., C. A., St, John, and Coleman, R. (2005). Did fertility go up after the Oklahoma City bombing? An analysis of births in metropolitan counties in Oklahoma, 1990–1999. Demography 42, 675–692. doi: 10.1353/dem.2005.0034

Rushton, J. P., Bons, T. A., and Hur, Y.-M. (2008). The genetics and evolution of the general factor of personality. J. Res. Pers. 42, 1173–1185. doi:10.1016/j.jrp.2008.03.002

Schafer, J. L. (1997). Analysis of Incomplete Multivariate Data. London: Chapman & Hall.

Waddington, C. H. (1953).Genetic assimilation of an acquired character. Evolution 7, 118–126. doi: 10.2307/2405747

Weiss, A., King, J. E., and Enns, R. M. (2002). Subjective well-being is heritable and genetically correlated with dominance in chimpanzees (Pan troglodytes). J. Pers. Soc. Psychol. 83, 1141–1149. doi: 10.1037/0022-3514.83.5.1141

West-Eberhard, M. J. (2003). Developmental Plasticity and Evolution. New York, NY: Oxford University Press.
